# Differential Diagnosis of the Diseases of the Skin

**Published:** 1887-11

**Authors:** 


**Differential Diagnosis of the Diseases of the Skin, for Students and Practitioners.** By C. W. Cutler, M. S., M. D., Assistant Attending Physician for Skin and Venereal Diseases, New York Hospital. New York and London: G. P. Putnam's Sons. 1887.

In this little book of 140 pages, the author presents, in tabulated form, the characteristic symptoms of such skin diseases as are most liable to be confounded one with another. We think the general practitioner will find the book of real service in enabling him to make a certain diagnosis in many cases which at first view may have puzzled him, and the student will find it a convenient aid in preparing for examination.

